# Investigation of CST7 and hsa-miR-4793-5p gene expression in breast cancer

**DOI:** 10.1016/j.bbrep.2024.101863

**Published:** 2024-11-01

**Authors:** Niloufar Sadat Kalaki, Mozhgan Ahmadzadeh, Mandana Dehghan, Venus Shahabi Rabori, Sima Davoudi, Hamed Afkhami

**Affiliations:** a Department of Cellular and Molecular Biology, Faculty of Biological Sciences, Kharazmi University, Tehran, Iran; b Student Research Committee, Shiraz University of Medical Sciences, Shiraz, Iran; c Department of Cardiology, Urmia University of Medical Sciences, Urmia, Iran; d Department of Clinical Oncology, Omid Hospital, Urmia University of Medical Sciences, Urmia, Iran; e Cellular and Molecular Research Center, Qom University of Medical Sciences, Qom, Iran; f Nervous System Stem Cells Research Center, Semnan University of Medical Sciences, Semnan, Iran; g Department of Medical Microbiology, Faculty of Medicine, Shahed University, Tehran, Iran

**Keywords:** Breast cancer, CST7, Hsa-miR-4793-5p, miRNA

## Abstract

Breast cancer (BC) presents as a worldwide challenge, known as the most frequently diagnosed cancer in women. In 2022, BC was diagnosed in 2.3 million women with 670,000 deaths globally. In this research, our objective was to examine the CST7 and has-miR-4793-5p gene expression in BC tumor tissues and adjacent normal tissues.

Using GSE57897 gene expression data from 422 BC samples and 31 breast samples from healthy controls which was based on the Platform GPL18722 (spotted oligonucleotide Homo sapiens microRNA (miRNA) array) in the Gene Expression Omnibus (GEO) and compare with miRNAs with a conserved target location on CST7 mRNA were found using databases. The study population included 60 fresh BC tissue samples and adjacent normal tissues as control. The Quantitative Real-Time PCR was used to evaluate the expression levels of CST7 and has-miR-4793-5p in the breast tissues.

The present study, found that CST7 and hsa-miR-4793-5p were significantly increased in tumoral tissues in compare to normal tissues. Further analysis revealed a remarkable association between CST7 and hsa-miR-4793-5p gene expression alteration. ROC curve analysis demonstrated high accuracy for CST7 expression in BC tumors. Comparison of gene expression between different stages and patient family history showed significant findings. Due to the high sensitivity and specificity of the expression changes of these two genes, they are suitable candidates for further investigations to be considered as part of a diagnosis and prognosis panel.

## Highlights

• The present study revealed that the expression of CST7 and hsa-miR-4793-5p were up regulated in breast cancer tumors and the expression of hsa-miR-4793-5p was associated with CST7 expression.
• probably hsa-miR-4793-5p exerts its regulatory effect on CST7 through other factors.
• Investigating the relationship between CTS7 and miR-4793-5p expression and how they affect the response to different treatment methods, could improve our comprehension of their functions in managing breast cancer.

## 1 Introduction

Breast cancer (BC) presents as a worldwide challenge, being the most frequently diagnosed cancer in women. In 2022, BC was diagnosed in 2.3 million women with 670,000 deaths globally. It can manifest in females of all age groups following the onset of puberty, regardless of geographical location, with a larger prevalence observed in older individuals [1]. Family history, age, estrogen, lifestyle, and reproductive factors are the most notable risk factors for BC, so that, approximately 25 % of all BC cases can be attributed to family history [2,3]. The development, treatment, and outcomes of BC are influenced by intrinsic genetic changes within tumors, categorizing them into molecular subtypes. Epigenetic modifications are important in the development of breast cancer because they perpetuate alternative gene activity states without altering the DNA sequence. These mechanisms regulate genomic stability, gene transcription, normal cell differentiation and cell growth. The three primary mechanisms of epigenetic alterations are non-coding RNAs (ncRNAs), histone modification, and DNA methylation, which collectively influence gene expression and transcription in specific cells and tissues, impacting cellular immunity. Furthermore, epigenetic changes are vital factors influencing the start and progression of tumors, involving local hypermethylation of CpG promoters of tumor suppressor genes, the loss of DNA methylation over the entire genome, the disruption of the ncRNA network, and extensive alterations in histone modification are all characteristic features of carcinogenesis [[4], [5], [6], [7]]. Previously, the role of micro RNAs in different stages of BC has been discussed and investigated, which led to the identification of their function in regulating the expression of different genes involved in different processes related to BC, such as angiogenesis, autophagy, apoptosis, drug resistance, etc [[8], [9], [10]].

Cysteine proteases are essential enzymes for protein degradation within lysosomes. Cathepsins, a type of cysteine protease, not only carry out general protein breakdown but also play pivotal roles in various cellular processes, including angiogenesis, apoptosis, invasion, and proliferation, Moreover, they are involved in antigen presentation, bone remodeling, and the maintenance of healthy skin. Certain members of the cathepsin protein family have been linked to tumor development and cancer metastasis [11,12].

The cystatin superfamily contains three different subfamilies that have specific characteristics. Cystatins in Family 1, such as cystatin A (CSTA) and B (CSTB), do not have disulfide bonds or signal peptides and are only active inside cells. Family 2 cystatins are proteins that are secreted and are around 115–120 amino acids long, and they have two interchain disulfide bonds. Family 3 cystatins, L- and H-kininogens, are complex glycosylated proteins found in the cytoplasm, with cystatin domains that resemble type-2 and a bradykinin moiety [13,14].

Family 2 of cystatins consists of seven members, namely cystatin SN (CST1), SA (CST2), C (CST3), S (CST4), D (CST5), E/M (CST6), and F (CST7). These cystatins belong to family 2 and are known as inhibitors of cysteine proteases present in different human fluids and secretions. They are believed to have a protective function. It has been previously observed that the levels of expression of family 2 cystatins are associated with biology of tumor, as well as tumor progression, and prognosis in colorectal, small-cell lung, and breast cancers [[15], [16], [17]].

The CST7 gene produces a glycosylated cysteine protease inhibitor that may have a function in immune control by blocking a specific target in the hematopoietic system. The protein's expression has been reported in several human cancer cell lines derived from malignant tumors [8].

Has-miR-4793-5p is a microRNA that is transcript from chromosome 3p21.31. it was described that has-miR-4793-5p associated with delayed cerebral infarction after aneurysmal subarachnoid hemorrhage [18].

CST7 is a protein-coding gene that has been associated with the progression of various cancers, including breast cancer, it has been suggested that CST7 may play a role in modulating the tumor microenvironment and influencing cancer cell behavior [19]. CST7 play a role in regulating the activities of cysteine proteases, which are enzymes involved in cell growth, invasion, and metastasis [20]. On the other hand, Utilizing GSE57897 gene expression data analysis showed hsa-miR-4793-5p expression was increased in BC patients, hsa-miR-4793-5p is a type of microRNA that has been implicated in breast cancer, in sillico studies showed hsa-miR-4793-5p has a binding site on CST7 gene. Therefore, both CST7 and hsa-miR-4793-5p are being researched for their potential roles in breast cancer development and progression.

In this research, our objective was to examine the CST7 and has-miR-4793-5p gene expression in BC tumor tissues and adjacent normal tissues.

## 2 Materials and methods

### Ethics approval

Every patient has given their informed permission and clinicopathological data in accordance with the regulations of the Ethics Committee of SBMU (IR.SBMU.MSP.REC.1399.270). The study protocol adhered to the principles outlined in the Declaration of Helsinki (Association, 2019).

### 2.1 Prediction of potential MiRNA interactions

Utilizing GSE57897 gene expression data from 422 BC samples and 31 breast samples from healthy controls which was based on the Platform GPL18722 (spotted oligonucleotide Homo sapiens microRNA array) in the Gene Expression Omnibus (GEO)(http://www.ncbi.nlm.nih.gov/geo/). MicroRNAs with a protected target site on CST7 mRNA were identified using the miRWalk (http://mirwalk.umm.uni-heidelberg.de/), TargetscanHuman8.0 database (www.targetscan.org), and miRTarBase (https://mirtarbase.cuhk.edu.cn/). Network analysis was carried out using Cytoscape v 3.9.0, and the validation of microRNAs was performed using the UALCAN database.

### 2.2 Patients and samples

Imam Hossein hospital in Tehran, Iran, collected 60 fresh BC tissue samples. Control breast tissue was taken from adjacent locations for examination. The study excluded patients who had undergone pre-operative chemotherapy or had additional malignancies. The tissue samples were rapidly frozen and stored at a temperature of −80 °C until the extraction of RNA.

### 2.3 Real-time PCR (QRT-PCR)

QRT-PCR was utilized to measure the expression levels of miR-4793-5p and CST7. Total RNA from patient samples was isolated via the Quick-DNA/RNA™ FFPE Kit (ZymoResearch). The assessment was conducted to evaluate the quality and concentration of the total RNA through electrophoresis in a 1.5 % agarose gel and by measuring the absorbance with Thermo Scientific™ NanoDrop 2000 to determine D260/D280 and D260/D230 ratios. The DNase I enzyme (Catalog number: EN0521; Thermo Fisher Scientific, United States) was employed for the purpose of eliminating DNA. The synthesis of CST7 cDNA was performed utilizing Oligo dT primers in accordance with the Geneall Kit manufacturer's guidelines. Quantitative real-time PCR (qRT-PCR) was conducted on a Rotor-Gene Q platform (Corbett Research, Sydney, AU) employing 2X QPCR Master Mix Syber Green (yektatajhiz, Iran). B-actin served as the internal reference for data normalization. The following primer sequences were employed for PCR amplification:

CST7:

Forward: 5′-GCGCTGTGATCATTTCTGGT-3′

Reverse: 5′-GGCTGTAGGAGTTGTGGAGT-3′

U6:

Forward: 5′-GCCTTTGCCGATCCGC-3′

Reverse: 5′-GCCGTAGCCGTTGTCG-3′

For miR-4793-5p analysis, initial miRNA polyadenylation was followed by reverse transcription using the BON-miR 1st-strand cDNA synthesis kit. Subsequent qRT-PCR was executed using BON-miR QPCR reagents (Stem Cell Technology, Iran), adhering to the manufacturer's protocol. Small Nucleolar RNA U6 was utilized as the internal reference for data normalization. Fold changes were calculated using the Pfaffl method [21].

### 2.4 Statistical analysis

The software Qiagen's REST 2009, developed in Hilden, Germany, was employed to analyze relative expression. The quantities of mRNA and miRNA in tissues or cell lines were standardized by comparing them to the levels of B-actin mRNA and U6, respectively. Comparisons were conducted between tumor or treatment replicas and nearby normal breast tissues or therapy disorganized cell lines. In order to comprehend the correlation between gene or miRNA expressions in a particular sample, the findings were displayed as the ratio of target genes to U6 expression, referred to as relative expression (REx). Three replicates of QRT-PCR were performed for each sample, and the findings were subsequently averaged. The expression data was subjected to statistical analysis using Graph Pad Prism 8.0. The *t*-test was employed to ascertain the rate of change in expression data, ROC curve analysis was used to evaluate sensitivity and specificity and AUC was used for reporting. The p-value less than 0.05 was deemed to be statistically significant.

## 3 Results

### 3.1 miR-4793-5p has potential to interaction with CST7

**Fig. 1:**
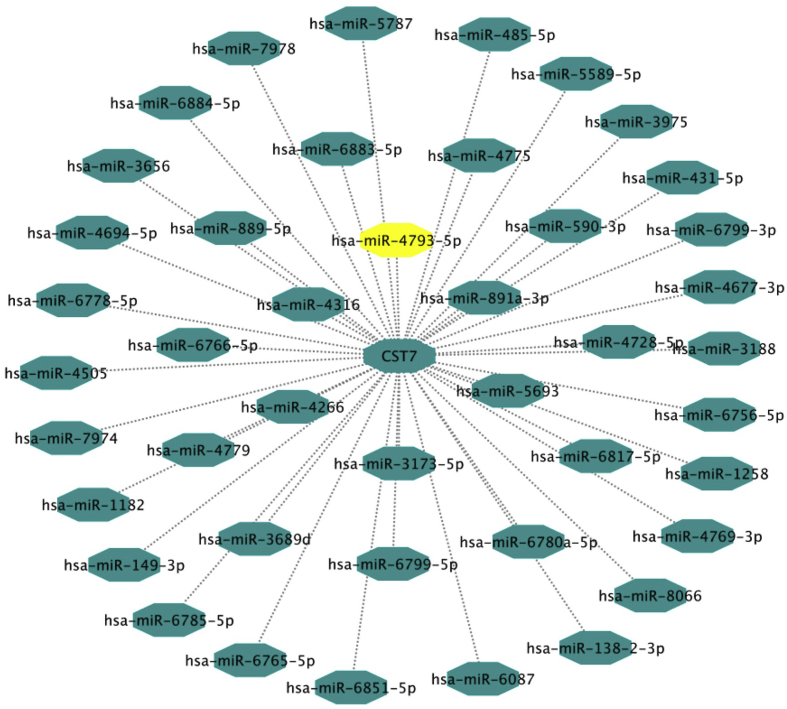
A) The network of miRNAs that predicted to interaction with CST7. The figure showed that miR-4793-5p has a binding site on CST7 gene. B) miR-4793-5p binding site at the 3′-UTR of CST7, ranging from base sites 40 to 46.

The results of GSE57897 dataset analysis showed that miR-4793-5p is altered in BC tumor tissue compared to normal tissue. MicroRNAs generally regulate target gene expression by directly binding to the 3′UTR region of the target gene's mRNA. Therefore, we used miRWalk, TargescanHuman8.0, and miRTarBase to predict the target microRNAs of CST7. The results Databases showed that that 45 miRNAs are related to CST7, miR -4793-5p has a binding site on CST7 gene (Fig. 1A). a bioinformatics analysis predicted a miR-4793-5p binding site at the 3′-UTR of CST7, ranging from base sites 40 to 46 (Fig. 1B).

**Fig. 2:**
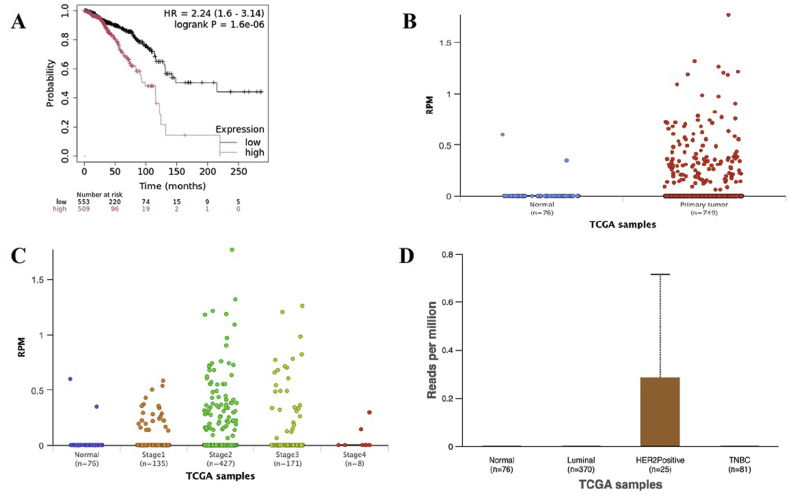
A)The survival analysis demonstrated that the BRCA patients with high expressions of hsa-miR-4793-5p (log-rank P = 1.6e-0.6) had significantly worse overall survival than those with low expressions; B) The expression level of hsa-miR-4793-5p between normal tissue and primary tissue in breast invasive carcinoma in the UALCAN database; the level of hsa-miR-4793-5p was remarkably upregulated in primary tissue in breast invasive carcinoma C) The expression level of hsa-miR-4793-5p between various stages in breast invasive carcinoma in the UALCAN database; hsa-miR-4793-5p expression was increased in stages 1,2 and 3 BC D) The expression level of hsa-C in breast invasive carcinoma based on subclasses in the UALCAN database, the expression level of hsa-miR-4793-5p based on subclass revealed the increase in Her2 positive group.

The survival data were analyzed by the Kaplan-Meier plotter database (http://kmplot.com/analysis/), which contained 1078 BRCA patients from TCGA database. According to Fig. 2A (Fig. 2A), the survival study revealed that BRCA patients with high levels of hsa-miR-4793-5p had significantly poorer overall survival compared to those with low levels (log-rank P = 1.6e-0.6). UALCAN database indicated that the level of hsa-miR-4793-5p was remarkably upregulated in primary tissue in breast invasive carcinoma (Fig. 2B), On the other hand, it was revealed that hsa-miR-4793-5p expression was increased in stages 1,2 and 3 BC (Fig. 2C), also the expression level of hsa-miR-4793-5p based on subclass revealed the increase in Her2 positive group (Fig. 2D).

### 3.2 CST7 and hsa-miR-4793-5p gene expression

**Fig. 3:**
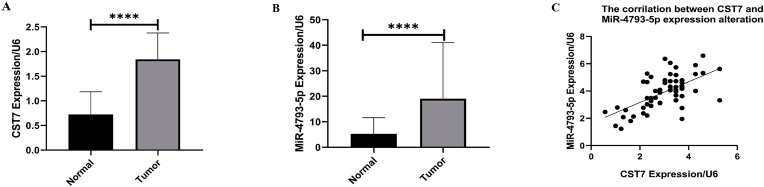
The expression of CST7 and hsa-miR-4793-5p. A) The CST7 expression was significantly upregulated in tumoral tissues. B) The hsa-miR-4793-5p expression was significantly upregulated in tumoral tissues. C) there was a positive correlation between CST7 and hsa-miR-4793-5p expressions.

**Fig. 4:**
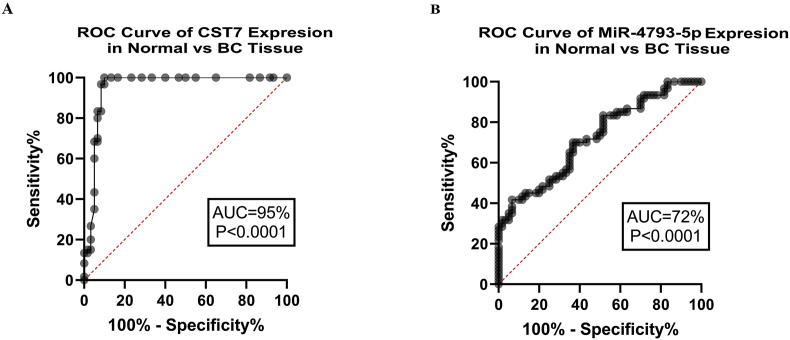
ROC curve analysis demonstrated an AUC of 95 % (P < 0.0001) for CST7 expression and 72 % (P < 0.0001) for hsa-miR-4793-5p in BC tumors.

The statically analysis revealed that CST7 and hsa-miR-4793-5p were substantially increased in tumoral tissues in comparison with normal tissues. CST7 and hsa-miR-4793-5p expression demonstrated 2.99 (P < 0.0001) and 3.91-fold (P < 0.0001) increase in tumoral tissues respectively (Fig. 3A and B). Further analysis described a significant association between CST7 and hsa-miR-4793-5p gene expression alteration (r = 0.557, P < 0.0001) (Fig. 3C). ROC curve analysis demonstrated an AUC of 95 % (P < 0.0001) for CST7 expression and 72 % (P < 0.0001) for hsa-miR-4793-5p in BC tumors (Fig. 4).

**Fig. 5:**
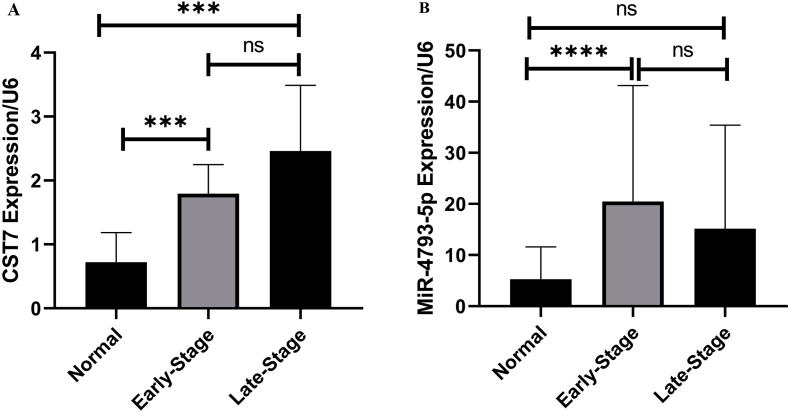
CST7 and MiR-4793-5P expression in different BC stages. A) The expression of CST7 was significantly upregulated in both early and late stages.

**Fig. 6:**
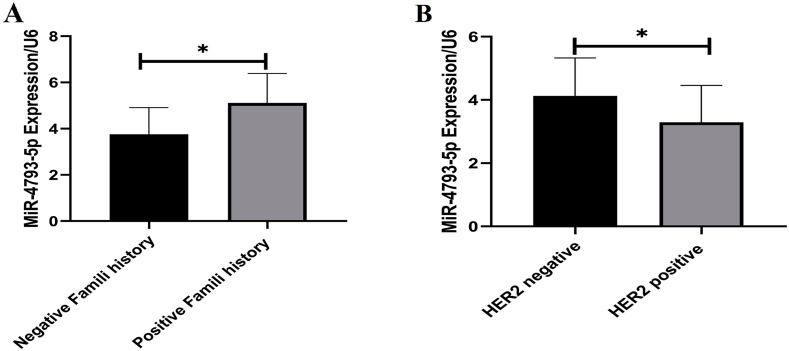
The MiR-4793-5p expression according to family history and Her2 receptor types. There was significant increase in the expression of miR-4793-5p in positive family history patients in comparison with negative family history patients.

Comparison of CST7 and hsa-miR-4793-5p gene expression between different stages showed that CST7 gene expression increased significantly in both early and late stages compared to normal tissue, while hsa-miR-4793-5p expression showed a significant increase in early stages (Fig. 5). Conversely, there was no significant differentiation in the expression of CST7 and hsa-miR-4793-5p in different age, metastasis, and tumor type (lobular or Ductal) groups. However, hsa-miR-4793-5pexpression shows a significant upregulation in the Her2 negative group in comparison with the Her2 positive group (P = 0.03), there was no significant differentiation in the expression of CST7 in these types. Also, there was a significant increase in the expression of hsa-miR-4793-5p in family-positive samples in comparison with family-negative samples(p = 0.01) (Fig. 6).

## 4 Discussion

BC is a very heterogeneous common disease, which has caused challenges in its treatment and overcoming. Therefore, the complete identification and understanding of the molecular mechanisms involved in this disease will be very efficient in the prevention, diagnosis, prognosis and treatment of the diseases [22,23]. One of the most important factors disrupting the normal pattern of the cell cycle and directing it towards the formation of cancer cells is the change in the expression of genes and their regulatory factors.

In the present study, we found that that CST7 and hsa-miR-4793-5p were significantly upregulated in tumoral tissues compared to normal tissues. Further analysis showed a significant association between CST7 and hsa-miR-4793-5p gene expression alteration. ROC curve analysis demonstrated high accuracy for CST7 expression in BC tumors. Comparison of gene expression between different stages and patient family history showed significant findings.

Previously, the role of Family 2 cystatins in different cancer was described. Maria Chimonidou and et al. found that the CST6 promoter is significantly methylated in the cell-free DNA (cfDNA) of breast cancer patients, but not in healthy persons [16]. The work conducted by Hünten and Hermeking shown that CST5 plays a crucial role in facilitating the suppression of tumors by p53 in colorectal cancer. Also it was suggested that of CST5 can decrease mesenchymal-epithelial transition (MET) induced by p53 [17]. Zhou et al. study shows that the level of CST7 was decreased in hepatitis B virus-related hepatocellular carcinoma. Moreover, it was disclosed that CST7 exhibited significant enrichment in cancer progenitors, liver cancer advancement, tumor evasion and tolerogenicity, late recurrence of liver cancer, and various subclasses of hepatocellular carcinoma [24].

On the other study, Yang et al. suggested CST7, a key family 2 cystatin subunit, is a potential prognostic biomarker for early-stage pancreatic ductal adenocarcinoma (PDAC). They reported that patients with a greater expression level of CST7 had a reduced risk in terms of overall survival [25]. The involvement of CST7 in cancer is intricate and potentially particular to certain types of cancer, as indicated by recent studies conducted on different types of cancer. The study conducted by Yao et al. on cervical cancer presents a compelling juxtaposition to our findings in breast cancer. Although we noticed an increase in CST7 expression in breast cancer tissues, Yao et al. discovered that enhancing CST7 expression decreased the proliferation and invasion capabilities of cervical cancer cells [26]. The disparity underscores the potential context-specific functions of CST7 in various forms of cancer and underscores the necessity for cancer-specific research. The study conducted by Yao et al. highlights the significance of the immune microenvironment in the advancement of cancer. It identifies CST7, along with IL1B and ITGA5, as three crucial genes that play a vital role in the evolution of cervical cancer [26]. This discovery reinforces our emphasis on CST7 in breast cancer and indicates that its significance may apply to several types of cancer. Further research should examine the involvement of CST7 in the infiltration of immune cells in breast cancer tissues and analyze possible connections between CST7, IL1B, and ITGA5 in relation to breast cancer. The ICI score system, established by Yao et al., offers a compelling technique that could be applied to breast cancer research [26]. Creating a comparable scoring system for breast cancer, which might include the evaluation of CST7 and miR-4793-5p expression, has the potential to offer significant understanding regarding the effectiveness of immunotherapy and the prediction of prognosis.

In addition, the utilization of single-cell sequencing data to examine the distribution of crucial genes among various cell types, as demonstrated by Yao et al., is a potent methodology that has the potential to be employed in forthcoming breast cancer research [26]. An examination of CST7 and miR-4793-5p expression at the individual cell level in breast cancer has the potential to offer more detailed understanding of their functions in particular cell types within the tumor microenvironment.

The relationship between miRNAs and the hallmark of cancer, particularly breast cancer, has a long history of regulation. In 2005, Lu et al. first documented the varying expression of miRNAs in breast cancer [27]. Since then, numerous studies have increasingly indicated the close association of miRNAs with the onset and progression of BC. It has been reported that miRNAs have two significant roles: they can act as either oncogenes (onco-miRNAs) or tumor suppressors [28].

The results of Insilco analyze and their confirmation with laboratory investigations introduced hsa-miR-4793-5p as a biomarker with diagnostic and prognosis properties. Before this, limited studies have been done on hsa-miR-4793-5p. The results suggest that this miRNA probably plays a role in the initiation and progression of breast cancer and reduces survival rate. Since the increase in the expression of this gene showed an association with the increase in the expression of the CST7 gene, it seems that this miRNA exerts its regulatory effect through other factors such as cellular microenvironment, epigenetic modifications, post-transcriptional regulatory elements, and other signaling molecules within the biological pathway. Moreover, genetic variations and environmental stimuli may also play a role in modulating this interaction. Understanding these factors will be crucial for comprehensively elucidating the regulatory network involving CST7 and hsa-miR-4793-5p. However, due to the high sensitivity and specificity of the expression changes of these two genes, they are suitable candidates for further investigations to be considered as part of a diagnosis and prognosis panel.

Considering the results of this study and the wider scope of miRNA investigation in breast cancer, various opportunities for further research become apparent. Targeting CST7 for therapeutic purposes shows promise due to its involvement in various biological processes. CST7 has been implicated in the regulation of immune responses and apoptosis, making it an attractive target for therapeutic interventions. Additionally, studies have suggested its potential role in cancer progression, indicating that inhibiting CST7 activity could be a viable strategy for cancer treatment.

Understanding the role of hsa-miR-4793-5p in various biological processes and its interaction with specific target genes could provide insights into potential therapeutic interventions. Modulating the expression or activity of hsa-miR-4793-5p through targeted approaches may offer a novel avenue for developing tailored treatments or interventions for certain conditions. Further research into the specific mechanisms and downstream effects of targeting hsa-miR-4793-5p is warranted to fully assess its therapeutic potential.

Targeting the interaction between hsa-miR-4793-5p and CST7 for therapeutic purposes in breast cancer holds great potential. This regulatory axis may play a critical role in breast cancer progression, and understanding its specific roles in the disease pathway could provide valuable insights for therapeutic interventions. Modulating the expression or activity of hsa-miR-4793-5p and CST7 through targeted approaches may offer a novel avenue for developing tailored treatments for breast cancer.

Prior to further investigation, it is imperative to carry out functional investigations in order to clarify the precise mechanisms by which miR-4793-5p controls CST7 and the impact of this interaction on the advancement of breast cancer. Furthermore, exploring possible therapeutic uses, such as utilizing miRNA mimics or inhibitors that target the miR-4793-5p/CST7 axis, could offer innovative treatment approaches. For f uture studies, it would be valuable to investigate the therapeutic potential of targeting the miR-4793-5p/CST7 axis in the context of breast cancer. This could involve designing experiments to manipulate the levels of miR-4793-5p and CST7 and observing the corresponding changes in cancer cell behavior, such as proliferation, invasion, and metastasis. Additionally, exploring the feasibility of using miR-4793-5p and CST7 as targets for novel therapeutic interventions, such as RNA-based therapeutics, could provide insight into potential treatment options for breast cancer. Furthermore, extending the investigation to assess the expression of miR-4793-5p and CST7 in other cancer types may uncover broader implications for their roles in cancer biology. Analyzing their expression profiles in different cancer tissues and cell lines could elucidate whether the regulatory effects observed in breast cancer are context-specific or if miR-4793-5p and CST7 play similar roles in other malignancies. Understanding the broader impact of miR-4793-5p and CST7 across varied cancer types could provide valuable insights for developing targeted therapies with potential applications beyond breast cancer.context of breast cancer progression. Further research is needed to elucidate the specific mechanisms and pathways through which hsa-miR-4793-5p exerts its regulatory effects on CST7. This study has some limitations, Additional large-scale cohort studies are required to confirm the predictive and diagnostic capabilities of CST7 and miR-4793-5p in various subtypes and stages of breast cancer. the study lacks functional validation experiments that could further elucidate the precise mechanisms by which hsa-miR-4793-5p may regulate CST7 expression. Additionally, investigating the relationship between CST7 and miR-4793-5p expression and how they affect the response to different treatment methods, such as targeted treatments and immunotherapies, could improve our comprehension of their functions in managing breast cancer. Furthermore, the interaction between CST7 and hsa-miR-4793-5p could potentially be influenced by various factors, Further research aimed at exploring these influencing factors is essential for a more thorough comprehension of the intricate relationships involving CST7 and hsa-miR-4793-5p.

## 5 Conclusion

The present study revealed that the expression of CST7 and hsa-miR-4793-5p were up regulated in BC tumors and the expression of hsa-miR-4793-5p was associated with CST7 expression. It was suggested that probably hsa-miR-4793-5p exerts its regulatory effect on CST7 through other factors.

## CRediT authorship contribution statement

**Niloufar Sadat Kalaki:** Writing – review & editing, Validation, Supervision, Project administration, Methodology. **Mozhgan Ahmadzadeh:** Writing – original draft, Software, Data curation, Conceptualization. **Mandana Dehghan:** Writing – original draft, Data curation, Conceptualization. **Venus Shahabi Rabori:** Writing – review & editing, Data curation. **Sima Davoudi:** Writing – review & editing, Data curation. **Hamed Afkhami:** Writing – review & editing, Validation, Supervision, Project administration.

## Ethics approval and consent to participate

Every patient has given their informed permission and clinicopathological data in accordance with the regulations of the Ethics Committee of SBMU (IR.SBMU.MSP.REC.1399.270). The study protocol adhered to the principles outlined in the Declaration of Helsinki (Association, 2019).

## Consent for publication

Not applicable.

## Availability of data and materials

Not applicable.

## Funding

No Funding

## Declaration of competing interest

The authors declare that they have no known competing financial interests or personal relationships that could have appeared to influence the work reported in this paper.

## Data Availability

Data will be made available on request.
